# RDP4: Detection and analysis of recombination patterns in virus genomes

**DOI:** 10.1093/ve/vev003

**Published:** 2015-05-26

**Authors:** Darren P. Martin, Ben Murrell, Michael Golden, Arjun Khoosal, Brejnev Muhire

**Affiliations:** 1 ^1^Department of Integrative Biomedical Sciences, Computational Biology Group, Institute of Infectious Disease and Molecular Medicine, University of Cape Town, Anzio Road Observatory 7549, Cape Town, South Africa; 2 ^2^Department of Medicine, University of California, San Diego, 9500 Gilman Dr., La Jolla, CA 92093, USA; 3 ^3^Department of Statistics, University of Oxford, 1 South Parks Road, OX1 3TG, Oxford, UK

**Keywords:** reassortment, horizontal gene transfer, lateral gene transfer, sequence analysis software

## Abstract

RDP4 is the latest version of recombination detection program (RDP), a Windows computer program that implements an extensive array of methods for detecting and visualising recombination in, and stripping evidence of recombination from, virus genome sequence alignments. RDP4 is capable of analysing twice as many sequences (up to 2,500) that are up to three times longer (up to 10 Mb) than those that could be analysed by older versions of the program. RDP4 is therefore also applicable to the analysis of bacterial full-genome sequence datasets. Other novelties in RDP4 include (1) the capacity to differentiate between recombination and genome segment reassortment, (2) the estimation of recombination breakpoint confidence intervals, (3) a variety of ‘recombination aware’ phylogenetic tree construction and comparison tools, (4) new matrix-based visualisation tools for examining both individual recombination events and the overall phylogenetic impacts of multiple recombination events and (5) new tests to detect the influences of gene arrangements, encoded protein structure, nucleic acid secondary structure, nucleotide composition, and nucleotide diversity on recombination breakpoint patterns. The key feature of RDP4 that differentiates it from other recombination detection tools is its flexibility. It can be run either in fully automated mode from the command line interface or with a graphically rich user interface that enables detailed exploration of both individual recombination events and overall recombination patterns.

## 1 Introduction

In many different groups of viruses, genetic recombination is an important evolutionary process that generates much of the genetic diversity upon which natural selection acts. Recombination patterns that are evident within the genomes of such viruses can reveal a great deal about their biology and evolution. Non-random patterns of sequence exchange between individuals within a species can provide direct evidence of geographical or host-range-imposed population subdivisions that prevent certain individuals from recombining (Lam et al. 2013; Monjane et al. 2014). Similarly, sequence exchange patterns between viruses in different species can reveal otherwise undetectable ecological links between some species and barriers between others (Beiko, Harlow, and Ragan 2005; Lefeuvre et al. 2010; Prasanna et al. 2010). The distributions of recombination breakpoints that are evident within virus genomes can also reveal details of the mechanistic and biochemical processes underlying recombination (Magiorkinis et al. 2003; Rohayem, Münch, and Rethwilm 2005; Lefeuvre et al. 2009; Dedepsidis et al. 2010; Simon-Loriere et al. 2010) and the selective forces that constrain the survival and proliferation of recombinants (Lefeuvre et al. 2007; Simon-Loriere et al. 2009; Golden et al. 2014; Woo, Robertson, and Lovell 2014). The epidemiological and/or ecological context of recombinants and the distributions of detected recombination breakpoints can also be crucial in identifying instances where recombinants have been artefactually generated in the laboratory (Boni et al. 2008; Han and Worobey 2011; Martin, Lemey, and Posada 2011; Tan et al. 2012; Lam et al. 2013).

Besides an interest in recombination itself, another important reason for analysing recombination patterns in virus genomes is to minimise the disruptive impact that recombination can have on other phylogeny-based analyses of molecular evolution (Schierup and Hein 2000b; Scheffler, Martin, and Seoighe 2006; Arenas and Posada 2010). Specifically, unaccounted for recombination events within a set of sequences can seriously undermine the accuracy of phylogenetic trees constructed from these sequences (Schierup and Hein 2000a; Posada and Crandall 2002). Therefore, it is often desirable to either exclude recombinant sequences or identify recombination breakpoint positions and focus analyses exclusively on those genome regions that are unbroken by these breakpoints prior to carrying out selection, molecular clock, phylogeographic, or any other analyses of virus genome sequences that may be misled by incorrectly inferred phylogenetic trees.

## 2 Detecting individual recombination events with RDP4

**Figure 1.**
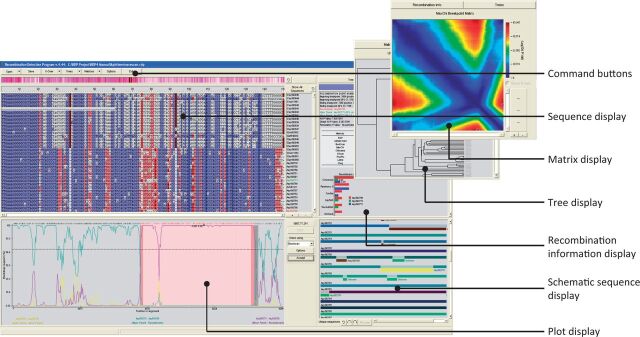
The main elements of the RDP4 program interface. The interface is split into four main resizable components: (1) a ‘zoomable’ sequence display that serves both as an alignment viewer and as a viewer of colour coded recombinant and parental sequences; (2) interchangeable tree/matrix/information displays that provide information on individual user-selected recombination events such as inferred breakpoint locations (and statistically plausible alternative locations), parental sequences (and phylogenetically plausible alternative parents), analysis warnings (such as if there is a high probability of recombinants and/or recombination breakpoints having been misidentified), and relative degrees of support by different analysis methods for detected recombination signals; (3) a schematic sequence display depicting colour-coded representations of the analysed sequences and the locations of detected recombination events; and (4) a plot display graphically illustrating the statistical evidence underlying the detection of individual user-selected recombination events.

RDP4 is a computer program that was developed with all of these applications in mind. Given a set of aligned nucleotide sequences, it identifies and characterises individual recombination events, providing detailed information on which sequences in the analysed dataset carry evidence of the same recombination event, the likely positions of recombination breakpoints, and the identities of sequences that are most closely related to the parental sequences. Key elements of the RDP4 program interface are illustrated in Fig. 1.

Crucially, RDP4 is able to perform recombination analyses without any need for predefined sets of non-recombinant reference sequences: a factor which makes it more generally applicable than many other available recombination analysis tools (see http://www.bioinf.manchester.ac.uk/recombination/programs.shtml; Martin, Lemey, and Posada 2011). RDP4 is able to do this using a range of fast and powerful heuristic recombination detection methods that sequentially test every combination of three sequences in an input alignment for evidence that one of the three sequences is a recombinant and the other two are its parents. Besides the original RDP method (Martin and Rybicki 2000), these methods include bootscan (Salminen et al. 1995), maxchi (Maynard Smith 1992), chimaera (Posada and Crandall 2001), 3seq (Boni, Posada, and Feldman 2007), geneconv (Padidam, Sawyer, and Fauquet 1999), lard (Holmes, Worobey, and Rambaut 1999), and siscan (Gibbs, Armstrong, and Gibbs 2000). Following the detection of a ‘recombination signal’ with these methods, RDP4 determines approximate breakpoint positions using a hidden Markov model, burt, and then identifies the recombinant sequence using the phylpro (Weiller 1998), visrd (Lemey et al. 2009), and eeep methods (Beiko and Hamilton 2006; Heath et al. 2006; see the manual that is distributed with RDP4 for a detailed account of how all of these methods work).

Having detected all of the recombination signals that are evident within an input alignment, RDP4 will then proceed to infer the minimum number of recombination events needed to account for these signals. It does so by sequentially disassembling identified recombinant sequences into their component parts (i.e., each recombinant sequence is split into two pieces) and iteratively rescanning the resulting expanded dataset until no further recombination signals are evident.

This fully exploratory approach means that, without any prior information, RDP4 can be used to characterise complex patterns of recombination such as those arising when recombination events occur between parental sequences that are themselves recombinant.

It is important to note, however, that there are also drawbacks to this approach. Primary among these is that when analysing datasets that contain large numbers of recombinant sequences, it can become very difficult for RDP4 to accurately identify the recombinants. Similarly, when numerous ancient recombination events have occurred such that multiple sequences in a dataset carry evidence of the same ancestral recombination events, RDP4 will often incorrectly attribute recombination signals arising from multiple different recombination events to a single ancestral event (i.e., it will under-count the number of recombination events evident within a dataset).

To partially rectify such deficits, RDP4 includes an array of tools which can be used to manually check, and correct if necessary, any perceived inference errors that the program has made. These tools are all accessible via a point-and-click graphical user interface and enable a user to directly test alternative hypotheses relating to the misidentification of recombination breakpoints, parental sequences, and groups of sequences sharing evidence of the same ancestral recombination events. Among others, these cross-checking tools include the following:

1. Multiple different phylogenetic tree construction methods that can be used to contrast phylogenetic signals in different parts of an alignment (such as on opposite sides of a recombination breakpoint).
2. Shimodaira–Hasegawa and approximately unbiased phylogenetic tree comparison tests (Shimodaira and Hasegawa 2001; Shimodaira 2002).
3. Matrix-based visualisations of the statistical plausibility of alternative breakpoint locations.
4. Statistical and phylogenetic tests that indicate the degree to which recombination signals that are detectable in two different sequences resemble one another.

## 3 Accounting for recombination during phylogenetics-based analyses

In cases where recombination is only being analysed with the intention of minimising its impact on other molecular evolution analyses, RDP4 can export sequence alignments in a multitude of formats either with recombinant sequences/fragments of sequences removed or with recombinant sequences split into their constituent parts. Such alignments will be stripped of all readily detectable evidence of individual recombination events and can then be used with other computer programs such as BEAST (Bouckaert et al. 2014) or HYPHY (Kosakovsky-Pond et al. 2005) to make more accurate estimates of evolutionary rates or less error-prone inferences of positive selection.

RDP4 can also be used to directly construct minimum evolution (with FastTree2; Price, Dehal, and Arkin 2010) and maximum-likelihood (with RAxML8; Stamatakis 2014) phylogenetic trees that account for the recombination events that it has detected. Specifically, it will construct trees using edited versions of the input alignment where fragments of sequence derived through recombination have either been removed altogether or have been re-added to the alignment as new sequences. Further, the program can carry out ‘recombination aware’ inferences of ancestral sequences using parsimony (with PHYLIP; Felsenstein 1989), maximum likelihood (with RAxML8; Stamatakis 2014), or Bayesian (with MrBayes3.2; Ronquist et al. 2012) approaches.

## 4 Tools for analysing overall patterns of recombination

**Figure 2.**
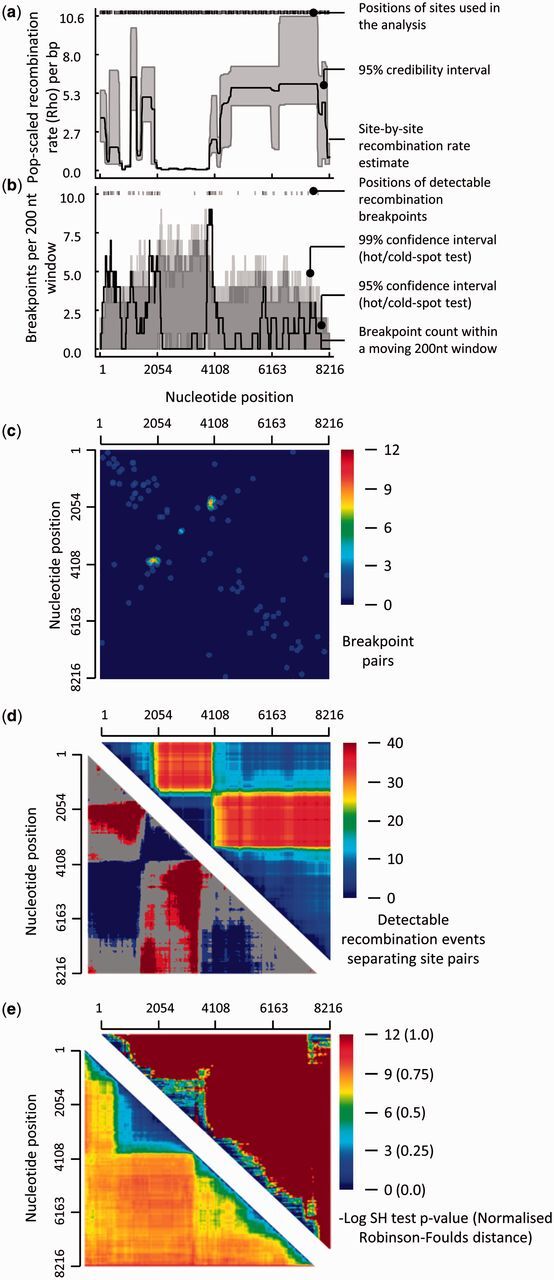
Examples of tools that are available in RDP4 for visualising overall patterns of recombination. The dataset examined here is the foot-and-mouth disease virus (FMDV) full genome dataset analysed in Heath et al. (2006; see the file, Example3(FMDV).rdp, that is distributed with RDP4). (a) Population-scaled recombination rate plots indicating variations in basal recombination rates across FMDV genomes and the presence of a likely recombination cold-spot between nucleotide positions ∼2000 and ∼4000. (b) Recombination breakpoint density plots indicating the presence of two recombination breakpoint hotspots at nucleotide positions ∼1900 and ∼4100. (c) Recombination breakpoint pair matrix. The yellow-red spot indicates that whenever a breakpoint occurs at nucleotide position ∼1900, there is a very strong tendency for a second breakpoint to occur at position ∼4100 (i.e., positions 1900 and 4100 are not only breakpoint hotspots: they are a breakpoint hotspot pair). (d) Recombination region count matrix. The top half of the matrix indicates that nucleotide site positions that are bounded by the recombination hotspots indicated in (b) tend to be co-inherited from the same parental virus (indicated by the dark blue triangle representing all pairs of sites between nucleotide positions ∼1900 and ∼4100). The bottom half of the matrix indicates site-pairs that are significantly more (in blue) or less (in red) frequently co-inherited during recombination than would be expected under random recombination. (e) Phylogenetic compatibility matrices illustrating the overall phylogenetic impacts of recombination in this FMDV dataset. Both the Shimodaira–Hasegawa (upper half) and Robinson–Foulds (lower half) compatibility matrices demonstrate that phylogenetic trees constructed for different parts of region ∼2000 to ∼4000 (indicated by the large blue-green triangles off the diagonals of both matrices) tend to be less different from one another than phylogenetic trees constructed from similarly sized portions of sequence sampled from elsewhere along the alignment (indicated by red-orange colours): these matrices therefore support the finding in (a) that there is a recombination cold-spot between nucleotide positions ∼2000 and ∼4000.

In cases where the underlying mechanistic or selective causes of detectable recombination patterns are of interest, RDP4 provides a range of useful tools including:

1. Tests for the presence of recombination hot- and cold spots (McVean et al. 2004, Heath et al. 2006; Fig. 2a–c).
2. Tests of purifying selection acting against recombination induced misfolding of either proteins (Voigt et al. 2002; Lefeuvre et al. 2007) or nucleic acid secondary structures (Golden et al. 2014).
3. Tests of association between recombination breakpoint locations and user-specified genome features (such as gene boundaries, the junctions between protein domains or nucleotides that are base-paired within secondary structures) (Lefeuvre et al. 2009; Simon-Loriere et al. 2010).
4. Tests for, and matrix-based visualisations of, the types of imbalanced coinheritance of nucleotide pairs that are expected to occur within recombinant genomes evolving under selection acting against the disruption of favourable epistatic interactions (Fig. 2d; Lefeuvre et al. 2009)
5. Phylogenetic incompatibility visualisations of the overall phylogenetic impacts of recombination within datasets (Fig. 2e; Jakobsen and Easteal 1996; Shimodaira and Hasegawa 2001; Simmonds and Welch 2006; Rousseau et al. 2007; Stamatakis 2014).

## 5 Operational limits

RDP4 can be used to productively analyse datasets containing up to 200 million nucleotides within 72 hours on a standard 2 GHz processor with 2 GB of RAM. Such datasets might, e.g., consist of sixty 3-Mb-long bacterial genome sequences, or 1,500 10-kb-long viral genome sequences. With default program settings, RDP4 can analyse 100 10-kb-long sequences in 10 minutes on a standard desktop computer.

## 6 Availability

RDP4 is available for free download from http://web.cbio.uct.ac.za/∼darren/rdp.html. It is distributed along with programs for generating (SDT; Muhire, Varsani, and Martin 2014) and aligning (IMPALE) datasets and an extensive manual that contains detailed descriptions of the various methods implemented in RDP4 and a step-by-step guide describing how best to use these. The manual and RDP4 site also contain information on how RDP4 can be run on Mac and Linux computers.
